# Modelling seasonal patterns of larval fish parasitism in two northern nearshore areas in the Humboldt Current System

**DOI:** 10.1038/s41598-020-79847-1

**Published:** 2021-01-12

**Authors:** Lissette D. Paredes, Mauricio F. Landaeta, Carlos Molinet, M. Teresa González

**Affiliations:** 1 Programa de Magíster en Ecología de Sistema Acuáticos, Facultad de Ciencias del Mar y Recursos Biológicos, Universidad de Antofagasta, Avenida Universidad de Antofagasta, 02800 Antofagasta, Chile; 2 Facultad de Ciencias del Mar y Recursos Biológicos, Instituto de Ciencias Naturales Alexander von Humboldt, Universidad de Antofagasta, Avenida Universidad de Antofagasta, 02800 Antofagasta, Chile; 3 Facultad de Ciencias del Mar y de Recursos Naturales, Universidad de Valparaíso, Avenida Borgoño, Reñaca, 16344 Viña del Mar, Chile; 4 Instituto de Acuicultura, Universidad Austral de Chile, Pelluco s/n, Puerto Montt, Chile

**Keywords:** Biodiversity, Ecological modelling, Ecology

## Abstract

Macro- and micro-environmental factors modulate parasite loads in fish, determining parasitic abundances, diversity, and interaction dynamics. In this study, seasonal variations in larval ectoparasites on fish larvae in the northern Humboldt Current System (HCS) were evaluated using a delta-gamma generalized linear model to predict their occurrence frequencies. Fish larvae were collected from two nearshore areas during austral spring–summer and autumn–winter. Only five (of 38) larval fish species were parasitized by copepods: *Gobiesox marmoratus*, *Ophiogobius jenynsi*, *Helcogrammoides cunninghami, Myxodes* sp., and *Auchenionchus crinitus*. A binomial model showed that the presence/absence of parasitized fish larvae varied among the fish species and their larval abundances, while a positive delta-gamma model showed that ectoparasite frequency varied among the seasons and fish species. Seasonal variations in parasitized fish larvae frequency could be associated with host and parasite reproductive processes, which are related to oceanographic features responsible for larval retention and subsequent higher infestation probabilities. Host length was positively correlated with ectoparasite length, suggesting early infection and combined growth until the detachment of the ectoparasite. Our results suggest that infestation patterns in larval fish species can be identified using delta-gamma models and that they respond to local (retention) and high-scale (HCS) processes.

## Introduction

Larval fish species are an important component of coastal ecosystems^1,2^, and they are highly vulnerable to environmental variation, predation, and parasitic infection^3^. However, previous research on the ecology of fish ectoparasites (parasites that infest the external surfaces of a host) has focused mainly on the biology of juvenile and/or adult fish interactions, and little is known about the interaction between larval ectoparasites and larval fish^4–7^.

The size relationships between hosts and parasites are well known for adult fish species, and parasitism is not generally associated with damage to these hosts^8,9^; however, the effect of parasitism on the health of larval fish species could potentially be high^10^. Although parasites are usually smaller than their hosts, their presence may have significant consequences regarding the physiological and ecological aspects of the development of larval fish species^11^. The presence of parasites could reduce the growth rate and health of larvae^4^, alter their organ functions and affect their ability to capture prey and avoid predators^12^.

Individuals at the larval stages of the ectoparasitic copepod of the genus *Trifur* (Copepoda: Pennellidae) have been reported to parasitize fish larvae from the families Clinidae, Engraulidae, Gobiidae, Gobiesocidae, Labrisomidae, Tripterygiidae, and Pinguipidae^5,13^. However, there are no records of *Trifur* spp. parasitizing adult individuals of these fish species. The basic life cycle of parasitic copepods comprises two phases, the naupliar and post-naupliar stages, prior to the adult stage, depending on the genus and taxon; individuals of each of these phases may parasitize a single host or more than one host^14^. Copepods of the Pennellidae family can utilize intermediate hosts to complete their larval development (copepodid stage, chalimus stage, and pre-metamorphic adult) and are able to detach from a host and swim while searching for their definitive host^15^.

Spatial and temporal variations in populations and communities of parasites are associated with different components: the micro-environment, which is the host body itself, and the macro-environment, which is the environment of the host^16^. Micro-environmental variations are recognized as the characteristics of the host, such as body size, host density, reproductive periods, food availability, and mortality rates^17^, while macro-environmental variations are recognized as the habitats of the host, which are associated with the natural changes in climate, environmental conditions (e.g., temperature, oxygen), and interspecific relationships that occur in every ecosystem^17,18^. Variations in these components may result in large differences in the abundance and diversity of parasites as well as different host-parasite dynamic interactions.

The Humboldt Current System is a highly productive marine ecosystem that is largely influenced by constant coastal upwelling that maintains a high level of biological productivity^19^ and supports a high abundance of larval fish species in the nearshore areas, especially in northern Chile^20^, which is characterized by a high retention of planktonic organisms and high food availability^21^. These local oceanographic conditions could favour the acquisition and retention of parasites in the water column and increase the probability of parasites encountering potential hosts. In this context, the objectives of this study were (1) to determine the magnitude of parasitism on larval fish species and (2) to predict the seasonal variations in ectoparasites affecting larval fish species from two nearshore areas of northern Chile using a delta-gamma generalized linear model (GLM) approach.

## Materials and methods

### Fieldwork

**Figure 1:**
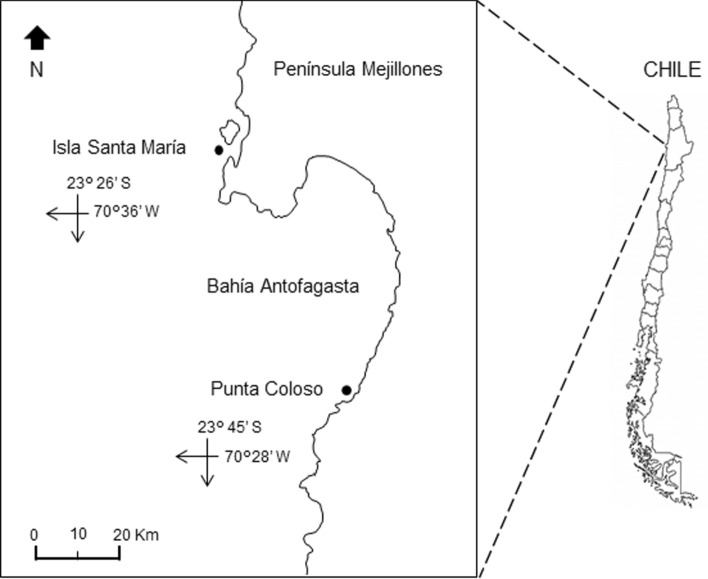
Study area of northern Chilean coast (Southeastern Pacific). The black points show the survey areas: Isla Santa María (ISM) and Coloso (COL).

The study was conducted in two nearshore areas (50 m offshore) around the Mejillones Península in the Antofagasta region of northern Chile, the Isla Santa Maria (23° 26′ S; 70° 36′ W) and Punta Coloso (23° 45′ S; 70° 28′ W) (Fig. 1). Five surveys were carried out in each area during the austral spring–summer season, between November 2013 and January 2014, and over the austral autumn–winter season, between May and August 2014.

Ichthyoplankton were collected from eight consecutive oblique tows from 10 m deep to the surface using a Bongo net (60 cm mouth diameter, 300 µm mesh size) equipped with a TSK flowmeter (The Tsurumi-Seiki Co. Ltd., Tsurumi-ku, Yokohama, Japan) to quantify the filtered volume. The tows continued for approximately 10–15 min each, and the volume of filtered seawater ranged between 1.42 and 680.15 m^3^ (mean ± standard deviation: 139.29 ± 116.83 m^3^). Half of the samples (*n* = 80) were fixed in 4% formalin buffered with sodium borate, and the other half (*n* = 80) were fixed with 96% ethanol in preparation for molecular analysis. The formalin-fixed samples were transferred to 96% ethanol after 24 h of the capture. ‘Ethical approval (code 048-2015) was given by Institutional Bioethics Committee for research with Animals (CIBICA)', “Dirección de Investigación of University of Valparaíso”, methods were carried out in accordance with relevant guidelines and regulations.

### Larval fish and parasite identification

All fish larvae were separated, counted, and classified to the lowest possible taxonomic level using the previous descriptions presented by Pérez^22^, Herrera^23^ and Zavala-Muñoz et al.^24^. Fish larvae and ectoparasites were photographed with an Olympus SZX7 camera and measured using MicroMetric software (Micrometrics Inc. 2009). The body length of each fish larvae was measured to the nearest 0.1 cm, from the tip of the upper maxilla to the tip of the notochord in pre-flexion larvae (notochord length) and to the base of the hypurals in flexion and post-flexion larvae (standard length). Each ectoparasites was measured in micrometres (µm) from the tip of the filament base to the end of the complex abdomen.

The ectoparasites were then separated and identified as belonging to the family Pennellidae and the genus *Trifur* based on morphological characteristics such as body shape, appendages, buccal structures, legs, genital complexes, and abdomens^5^. Additionally, molecular analysis based on the Cytochrome Oxydase I (COI) gene was performed to support the identification of the larval parasites collected from the larval fish species. DNA samples were extracted, and a PCR analysis was conducted, following the protocol used by González et al.^25^. The PCR products were sequenced by Macrogen, Inc. (Seoul, South Korea; http://www.macrogen.com) and BLAST was used to identify the genus *Trifur* through the comparison of the sequences obtained in this study with those available in GenBank.

### Data analysis

The prevalence of ectoparasites (= percentage of parasitized fish larvae per survey) and the mean intensity (mean number of ectoparasitic individuals considering only parasitized fish larvae per survey)^26^ were calculated for each larval fish species per area and survey.

For each fish species, Student’s t-tests were used to compare the body length of parasitized and unparasitized fish larvae collected during autumn–winter (the season in which the most parasitized fish larvae were recorded). For these comparisons, we used all recorded parasitized fish larvae and a subset of unparasitized fish larvae that were randomly selected. Spearman correlations (r_s_) were calculated to evaluate the association between parasite length and parasitized fish larvae length^27^.

The frequency of parasitized fish larvae was evaluated, taking into account the following variables: larval abundance of fish species, season (spring–summer and autumn–winter), fish species (*Auchenionchus crinitus, Gobiesox marmoratus*, *Helcogrammoides cunninghami, Myxodes* sp. and *Ophiogobius jenynsi*), and areas (Isla Santa María and Punta Coloso). The large number of zero samples made it impossible to apply simple mean comparison approaches to analyse the frequency of parasitized fish larvae. If zero samples had been included, normal assumptions would have been violated. If zero samples had been ignored, a relevant portion of the information would had been lost. Hence, we selected a delta-distribution approach to incorporate into the general linear model (GLM) framework to estimate parameters (sensu^28^), based on the Aitchison-Pennington method^29,30^. Here, the observable positive density (*d*_*p*_) was considered a random variable with a spike of probability mass at the origin. This means that ($\hat{\overline{d}}_{p}$) was calculated by ignoring the null observations, which were used to produce an independent estimate of the probability of larval presence in a sample ($\hat{\overline{p}}$*)* and then combined into a corrected density estimate ($\hat{\overline{d}}_{c}$), obeying the following relationships:

$$\hat{\overline{d}}_{c} = \hat{\overline{p}} \cdot \hat{\overline{d}}_{p} \cdot v(\hat{\overline{d}}_{c} ) = \hat{\overline{p}}^{2} \cdot v(\hat{\overline{d}}_{p} ) + v(\hat{\overline{p}}) \cdot \hat{\overline{d}}_{p}^{2} .$$

The positive density means were calculated through a general linear model procedure by assuming a gamma distribution^28^, a binomial negative distribution and a Poisson distribution^31^ of errors and using a log-link function, the adjustments of which were compared using the Akaike information criterion^32^. The gamma distribution of errors was selected because of its greater flexibility in accommodating non-normal datasets^33^. The presence or absence (prevalence) of parasitized fish larvae in our samples was analysed as a dichotomous variable (0/1) and used to estimate $\hat{\overline{p}}(d > 0)$ using a general linear mixed model procedure. For this, we assumed a binomial distribution of errors and used a logit-link function.

Since *d*_*c*_ was the product of two estimates generated by two independent sub-models ($\hat{\overline{p}}(d > 0)$ and $\hat{\overline{d}}_{p}$), there was no conventional F-test suitable for the simultaneous testing of alternative hypotheses. Hence, we used a 2-step iterative selection procedure to choose among alternative models: (i) a forward selection procedure was used to add and retain explanatory variables by providing significant regression coefficients (t-test) for at least one of the two sub-models; and (ii) competing/related explanatory variables (such as the categorical variable “season” and the continuous variable “larval fish abundance”) were evaluated. The most informative model was selected using the Akaike information criterion (AIC)^32,34^. All statistical procedures were carried out with R 3.3.3 (The R Development Core Team, 2017) using the car^35^, MASS, nlme, and lme4^36^ packages.

## Results

**Figure 2:**
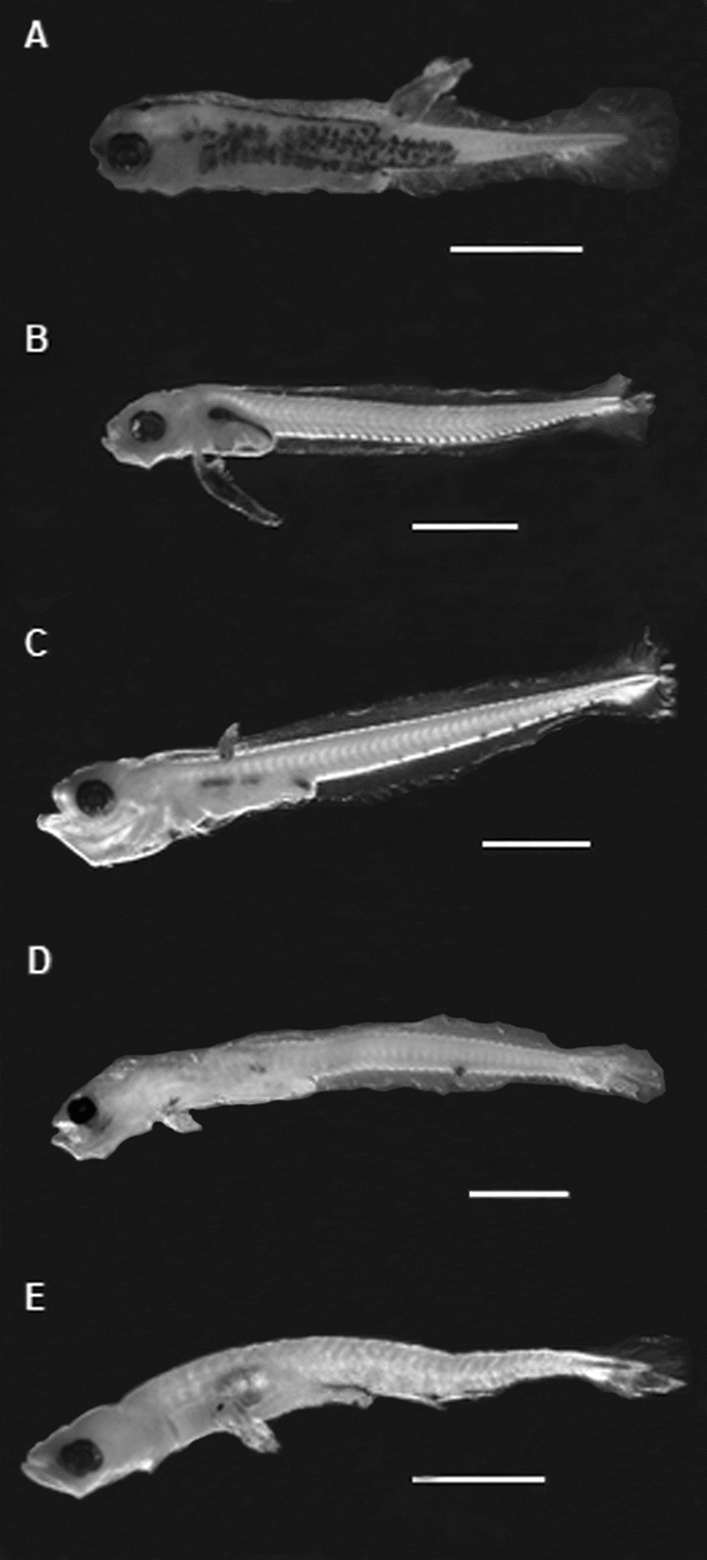
Photography of parasitized fish larvae species recorded in area of study. (**A**) *Gobiesox marmoratus*, (**B**) *Helcogrammoides cunninghami*, (**C**) *Auchenionchus crinitus*, (**D**) *Myxodes* sp., (**E**) *Ophiogobius jenynsi*.

During the whole period of the study, 47,628 fish larvae belonging to 38 taxa were identified (Supplementary Table S1). Of these, 369 larvae belonged to five fish species (Fig. 2) of the families Tripterygiidae, Gobiesocidae, Labrisomidae, Clinidae and Gobiidae that were parasitized by larval copepods resembling a species of *Trifur* (Pennellidae, n = 382). The prevalence of ectoparasites was different in each survey from both seasons and areas. In Isla Santa María, the prevalence varied from 0 to 16.7% (n = 359) in spring–summer and between 0 and 10.8% in autumn–winter (n = 18,170). In Punta Coloso, the prevalence was between 0% and 26.1% in spring–summer (n = 142) and between 0% and 14.3% in autumn–winter (n = 872) (Table 1). The larval fish *H. cunhinghami* (n = 9833) and *A. crinitus* (n = 8231) were the most abundant among the parasitized species, followed by *G. marmoratus* (n = 796), *O. jenynsi* (n = 411), and *Myxodes* sp. (n = 262) (Table 1). The body length of the larval of these species did not differ between unparasitized and parasitized fish larvae (all P > 0.05), except for *A. crinitus* (Table 2). The lengths of the ectoparasites were positively correlated with the lengths of individuals of *A. crinitus*, *G. marmoratus,* and *Myxodes* sp. from Isla Santa María and with the lengths of individuals of *G. marmoratus* from Punta Coloso (Table 2).

**Table 1:** Prevalence and mean intensity of larval copepods (*Trifur* sp.) found on five different fish larvae species collected from Isla Santa María and Punta Coloso during austral spring–summer (1–5) and autumn–winter (6–10) in northern Chile.

Areas	Survey	Larval fish species				
		*Auchenionchus crinitus*	*Gobiesox marmoratus*	*Helcogrammoides cunninghami*	*Myxodes* sp.	*Ophiogobius jenynsi*
Isla Santa María	1	[3]	[0]	[0]	[1]	
	2	[8]	16.67 (1) [6]	[28]	[2]	
	3	1.79 (1) [56]	12.5 (1) [8]	4.76 (1) [63]	– (1) [1]	
	4	3.57 (1) [28]	[24]	[13]	[3]	
	5	[18]	[3]	[2]	[0]	
	6	0.55 (1) [1267]	3.51 (1) [57]	6.50 (1.04) [1492]	2.24 (1) [134]	2.42 (1) [124]
	7	0.57 (1) [353]	[11]	0.94 (1.33) [320]	[15]	12.5 (1) [8]
	8	1.40 (1.05) [1502]	1.23 (1) [81]	3.28 (1.07) [1372]	1.98 (1) [101]	6.40 (1.22) [172]
	9	[642]	1.22 (1) [82]	2.18 (1) [642]	[5]	[17]
	10	0.63 (1) [3949]	10.81 (1) [74]	1.23 (1.01) [5843]	[0]	4.76 (1) [21]
Punta Coloso	1	[3]	7.69 (1) [13]	[2]		
	2	[4]	26.09 (1) [23]	[0]		
	3	[3]	4.55 (2) [44]	[0]		
	4	[4]	13.64 (1) [22]	[1]		
	5	[0]	[0]	[0]		
	6	[1]	[9]	[1]		[0]
	7	[5]	[9]	[1]		[1]
	8	[80]	[81]	[1]		[30]
	9	[114]	11.6 (1.4) [138]	[12]		14.29 (1.6) [28]
	10	1.50 (1) [200]	3.6 (1) [111]	2.50 (1) [40]		10.0 (1) [10]

In square parenthesis indicate number of larval fish collected in the respective survey.

**Table 2:** Body length ranges of unparasitized and parasitized fish larvae species, parasitized body size and correlation coefficients between parasitized fish larvae length and ectoparasite length per each area (Isla Santa María and Punta Coloso).

Species	Range unparasitized fish larvae	Range parasitized fish larvae	Range parasite size	Isla Santa María	Punta Coloso
				r	r
*Helcogrammoides cunninghami*	2.9–9.1 (109)	3.8–10.1 (235)	0.28–1.72	0.626 (234)	1.000 (1)
*Auchenionchus crinitus*	3.5–9.8 (151)	3.0–8.1 (61)	0.26–2.88	0.740* (58)	1.000 (3)
*Gobiesox marmoratus*	3.1–7.4 (132)	1.88–6.6 (46)	0.18–2.35	0.652* (14)	0.871* (32)
*Myxodes* sp.	2.2–5.5 (37)	4.1–6.1 (6)	0.55–2.08	0.982* (6)	–
*Ophiogobius jennynsi*	2.2–4.2 (35)	2.3–7.5 (21)	0.54–0.97	0.700 (16)	0.684 (5)

*Significant at P < 0.05.

**Figure 3:**
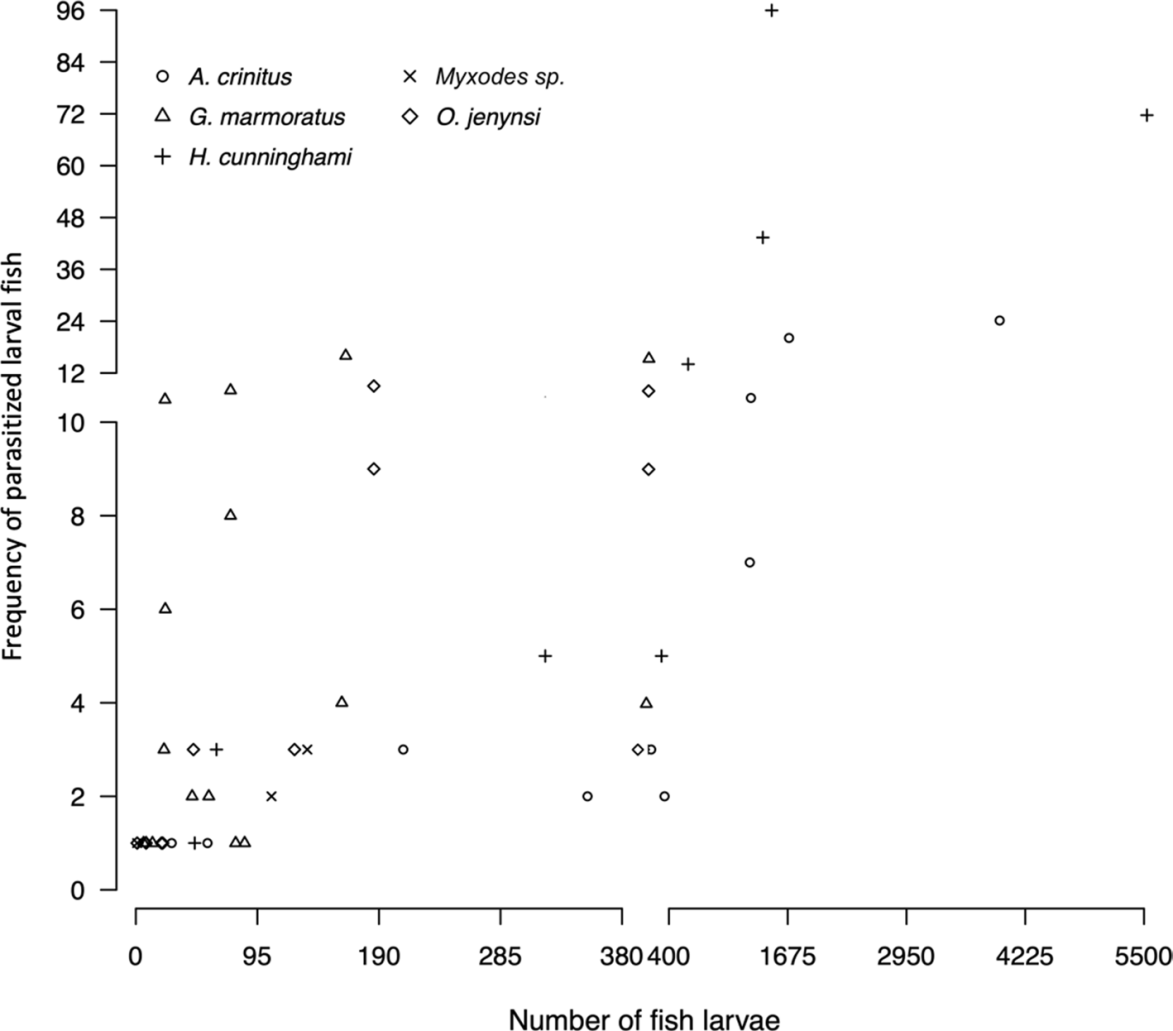
Relationship between larval fish abundance and frequency of parasitized fish larvae per survey, according to fish species.

The Akaike information criterion (AIC), applied for the three GLM models tested to explain the frequency of parasitized fish larvae [delta-gamma; delta-negative binomial and Poisson zero-inflated (delta-Poisson)], showed that the lowest AIC value was obtained with the delta-gamma generalized linear model (AIC = 279.4; Table 3). This model showed that the presence/absence of parasitized fish larvae was affected by the fish species and larval fish abundance (Table 4; Fig. 3), while the gamma model applied to determine the positive frequency of parasitized fish larvae showed effects of fish species and season (Table 4), with multiple comparisons showing that significant differences were present between *H. cunninghami* and *G. marmoratus* (Table 5).

**Table 3:** Selection of the most informative model (using the Akaike information criterion) to explain the variability of the frequency of parasitized fish larvae species considering as predictors the variables larval fish abundance, season (Spring–Summer and Autumn–Winter), fish species (*H. cunninghami*, *A. crinitus*, *G. marmoratus*, *Myxodes* sp., *O. jenynsi*), and area (Isla Santa María and Punta Coloso).

Type of model	Model	Akaike information criterion (AIC)
Generalized linear model delta-Gamma	Parasite frequency = Larval fish abundance + Season + Fish species + Area	279.4
Generalized linear model negative binomial Zero-inflated (delta-Negative binomial)	Parasite frequency = Larval fish abundance + Season + Fish species + Area	286.4
Generalized linear model Poisson Zero-inflated (delta-Poisson)	Parasite frequency = Larval fish abundance + Season + Fish species + Area	384.2

**Table 4:** Analysis of deviance for the presence and absence (GLM Binomial) and positive frequency (GLM Gamma) of ectoparasite on five fish species (*H. cunninghami*, *A. crinitus*, *G. marmoratus*, *Myxodes* sp., *O. jenynsi*), two areas (Isla Santa María and Punta Coloso) and two seasons (Spring–Summer and Autumn–Winter).

	Likelihood ratio	Degree of freedom	P (Chi-sq)
**Binomial model for presence/absence observations**			
Larval fish abundance	18.0349	1	2.17E−05*
Fish species	16.7486	4	0.002163*
Area	0.0613	1	0.804442
Season	2.3201	1	0.127708
Fish sp: Area	0.3967	3	0.94093
**Gamma model for positive observations**			
Number of fish larvae	1.3225	1	0.2501
Fish species	20.778	4	0.0003*
Area	1.2838	1	0.2571
Season	13.8274	1	0.0001*
Fish sp: Area	11.6522	3	0.008*

*Significant at P < 0.05.

**Table 5:** Multiple comparison for the variable fish species for the positive frequency of parasitized fish larvae, applying a Gamma Model (see Table 1).

Linear hypotheses	Estimate	Std. Error	z value	Pr (|z|)
Gm–Ac = 0	0.1442	0.0996	1.448	0.4347
Hc–Ac = 0	− 0.08701	0.05193	− 1.676	0.3047
Oj–Ac = 0	0.25175	0.16671	1.51	0.3969
**Hc–Gm = 0**	− 0.23121	0.08689	− 2.661	0.0327*
Oj–Gm = 0	0.10755	0.18068	0.595	0.9245
Oj–Hc = 0	0.33876	0.15945	2.125	0.1264

Hc, *H. cunninghami*; Gm, *G. marmoratus*; Ac, *A. crinitus*; Oj, *O. jenynsi.*

*Significant at P < 0.05.

**Figure 4:**
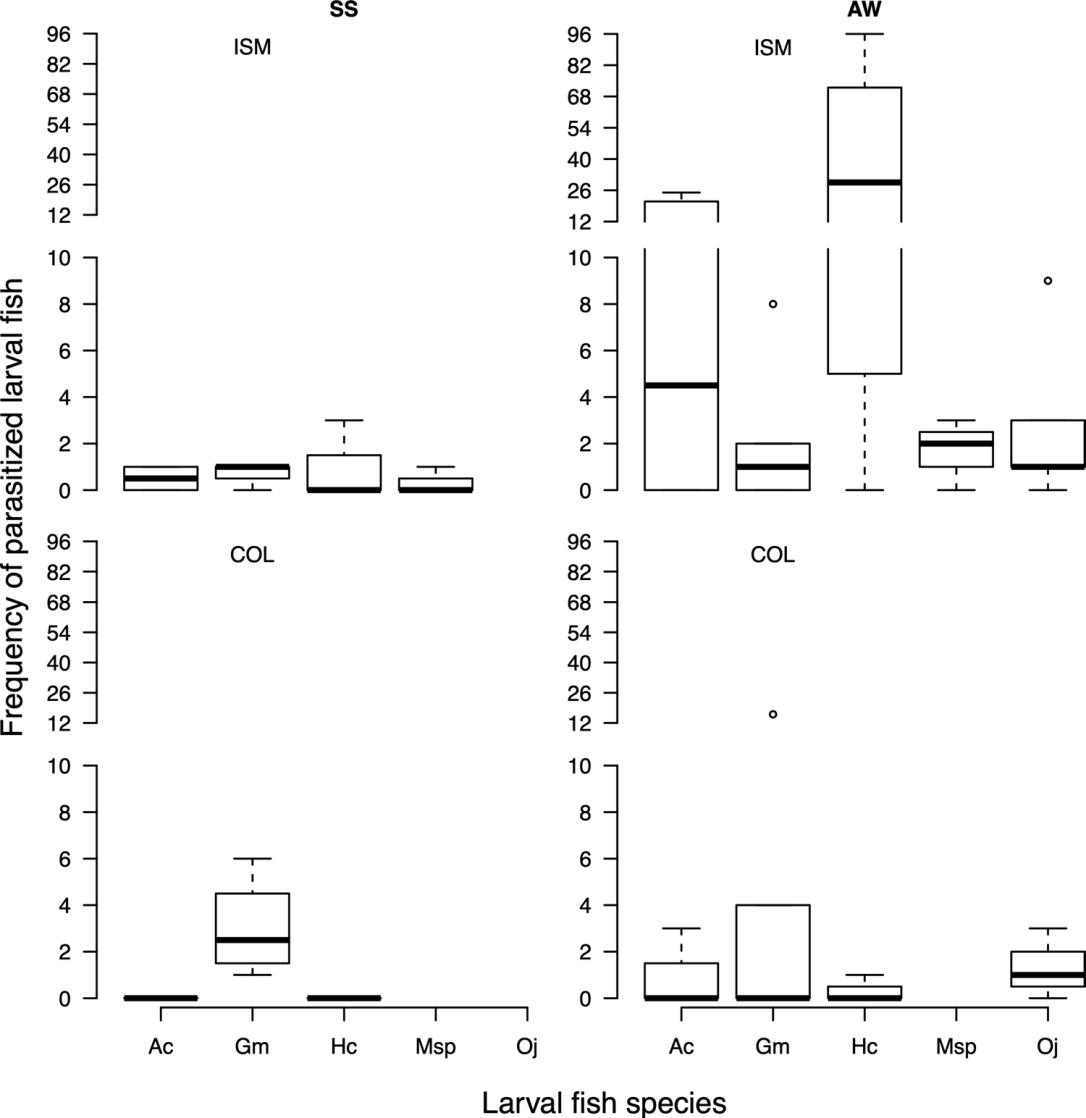
Boxplot of frequency of parasitized fish larvae observed in 5 fish species in two areas during the spring–summer and autumn–winter seasons. Bold line represents the median, inferior limit and superior box limit represent quantile 1 and 2 respectively, error bars represent maximum and minimum values. Ac, *Auchenionchus crinitus*; Gm, *Gobiesox marmoratus*; Hc, *Helcogrammoides cunninghami*, Msp, *Myxodes* sp.; Oj, *Ophiogobius jenynsi*.

The frequency of parasitized fish larvae was higher in Isla Santa María during autumn–winter, with *H. cunninghami* being the species most frequently parasitized by *Trifur* sp., followed by *A. crinitus* and *G. marmoratus* (Fig. 4). In contrast, in Punta Coloso, the frequency of parasitized fish larvae was lower than that in Isla Santa María, with *G. marmoratus* being the most parasitized fish species in both autumn–winter and spring–summer (Fig. 4).

**Figure 5:**
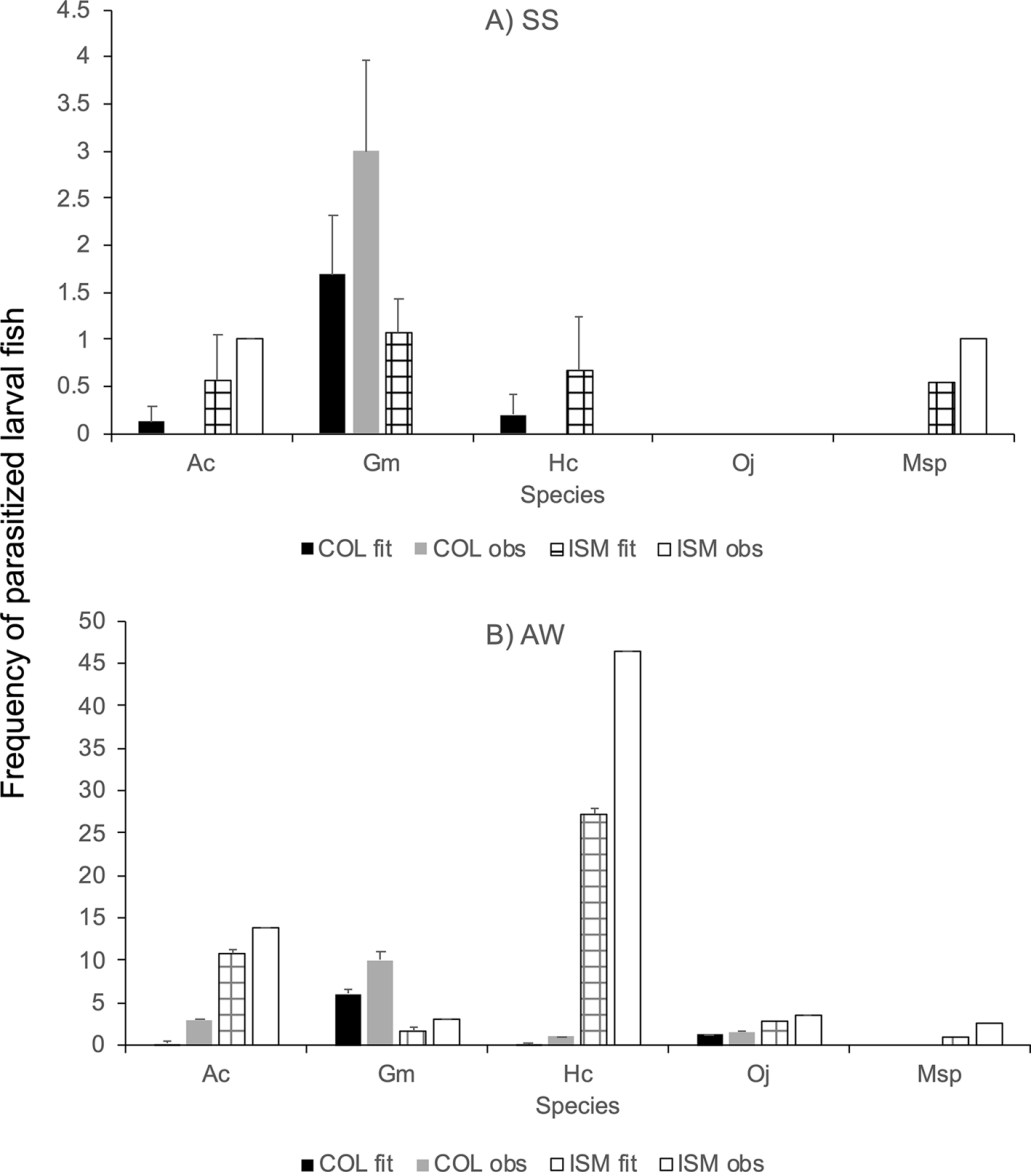
Observed and predicted frequency of parasitized fish larvae on 5 fish species in two areas during seasons. (**A**) SS (spring–summer) and (**B**) AW (autumn–winter). Error bars show the standard error. Ac, *Auchenionchus crinitus*; Gm, *Gobiesox marmoratus*; Hc, *Helcogrammoides cunninghami*; Msp, *Myxodes* sp.; Oj, *Ophiogobius jenynsi*.

The GLM predicted that ectoparasites would be very rare in both areas during the spring–summer season; only *G. marmoratus* would be expected to host *Trifur* sp. in Punta Coloso and Isla Santa María in spring–summer (Fig. 5a). Moreover, the model predicted that during the autumn–winter season, larvae of *H. cunninghami* and *A. crinitus* would be parasitized only in Isla Santa María, and larvae of *G. marmoratus* were expected to be parasitized in Punta Coloso (Fig. 5b).

## Discussion

Studies of copepods parasitizing fish larvae around the world are scarce. However, a few studies have reported parasitic larvae of the family Pennellidae on larval fish belonging to different families^4,5,7^. Our study adds more data to the record of such ectoparasites; this work documented ectoparasitic pennellid larvae (*Trifur* sp.) infesting several larval fish species with prevalence values varying between 0 and 26% per survey, according to season and area. Additionally, this is the first report of parasitized fish larvae belonging to the genus *Myxodes* in northern Chile.

The five larval fish species that were parasitized (*H. cunninghami*, *A. crinitus*,* G. marmoratus, Myxodes* sp. and *O. jenynsi*) characteristically live in shallow intertidal-subtidal zones as adults, but their planktonic larvae are located off the coast and remain there for a wide range of time periods (called the planktonic larval duration, PLD), extending from days to months, before reaching the subtidal and intertidal zones. The length of the PLD depends on the species and is relatively long because of the cold waters of the HCS: the PLD lasts 61–98 days for *G. marmoratus*, 78–115 days for *H. cunninghami*, 56–92 days for *A. crinitus*, 69–118 days for *Myxodes viridis*^37^, and 19–30 days for species of the family Gobiidae, such as *O. jenynsi*^38^. Grutter et al.^39^ suggested that the length of time a larval fish spends in the plankton can increase its chance of parasitic infection. However, a recent study indicates that the presence and intensity of parasites would not be related to the age or size of the fish larvae^40^. Along the HCS, a combination of low seawater temperatures (caused by upwelling events), thermal fronts (which increase coastal retention^21^), slow larval fish growth rates (typically between 0.08 and 0.22 mm day^−1^^41,42^) and long PLDs (~ 2 months) may account for copepod infestation on larvae of cryptobenthic fish. Other larval fish species with longer PLDs, such as *Scartichthys viridis* (72–124 days) and *Calliclinus geniguttatus* (83–135 days)^37^, however, were unparasitized during all study periods (see Supplementary Table S1), probably because they are advected offshore during their pelagic life, and the chance of parasitism is lower in oceanic waters^20,43^. Moreover, considering that (i) larval ectoparasites respond to non-specific visual stimuli, and (ii) spend approximately 2 days in the water column before detecting their host^16^, visual cues from the host would not be sufficient to explain why only five species of the total larval fish assemblage were parasitized. Therefore, it is possible that chemical cues generated by the potential host species are involved in encounters between larval parasites and fish larvae, as has been demonstrated for other parasitic copepods^44^.

Biotic and abiotic conditions influence the abundance of ectoparasite populations, which in turn can be directly associated with host population density^9,45^. In this study, a higher frequency of parasitized fish larvae was recorded in the austral autumn–winter season (May–August) when the larval abundances of the five fish species were significantly higher than those in spring–summer. However, at the latitudes of the central Chilean coast (33° S–36° S), the peak number of fish larvae parasitized by pennellids has been recorded during warm periods such as summer and early autumn; higher densities of fish larvae are also associated with those seasons^15,43^. The temporal lag in the prevalence of parasitic copepods between latitudes could be associated with the reproductive cycle of copepods, which begins when the water temperature increases^46^. Unlike at central latitudes, where the water temperature varies between 10 °C in winter and 16 °C in spring–summer, the northern latitudes of the Chilean coast are characterized by warmer water throughout the year (16–20 °C)^19^. Therefore, it is plausible that the reproductive period of the parasitic copepods in northern latitudes is extended because the water temperature remains over 15 °C year-round, allowing copepod to expulse eggs even during the autumn–winter season.

The frequency of parasitized fish larvae in Isla Santa María was higher than that in Punta Coloso. These local differences in larval infestation could be associated with oceanographic conditions, as well as the topography of these nearshore areas and the habitats that they provide. In this context, Isla Santa María, a semi-closed bay of rocky reefs with kelp forests of *Lessonia trabeculata* and *Macrocystis integrifolia* and high nutrient inputs^20,47^, provides a favourable habitat with high planktonic biodiversity, food availability, and shelter for optimizing the growth and survival of fish larvae^48^; promoting the acquisition and retention of parasites in the water column. While Punta Coloso is an open coastal area with an ocean bottom that mainly comprises sand, gravel, and mud as well as patches of *L. trabeculata* kelp^24^, in addition to the presence of an upwelling plume and a cyclonic eddy in front of the bay^21^; conditions that promote the offshore transport of planktonic organisms, decreasing the probability of host-parasite encounters.

Parasites in fish larvae could have detrimental impacts on the nutritional and immune response as well as mechanic effects can be caused by parasites via their own body weight^11^. All of these levels could affect the larval growth prior to settlement in fish species^7^. In our study, positive correlations between parasite size and larval fish size were recorded in *A. crinitus*, *G. marmoratus*, and *Myxodes* sp., reflecting that, at least in northern latitudes of the HCS, ectoparasites infest the larval fish species early in the planktonic larval stage, and both grow together until the larval copepod detaches and looks for its next host, suggesting that there are no lethal or detrimental effects on fish larvae survival.

In summary, our results suggest that infestation patterns in larval fish species can be identified using delta-gamma models and that the infestation patterns respond to local (retention) and high-scale (HCS) processes. GLMs (bimodal and delta-gamma) identified the importance of the fish species identity and its abundance on ectoparasite prevalence, predicting that a higher number of *H. cunninghami* larvae would be parasitized in the autumn–winter season mainly in Isla Santa María, while fewer than five larvae individuals of *G. marmoratus* and *A. crinitus* would be parasitized in the Isla Santa María and Punta Coloso areas during spring–summer. Additionally, our results show the importance of local environmental conditions such as water temperature and thermal fronts (which increase the coastal retention of larval parasites and their hosts) in northern latitudes, which favour parasite reproduction by extending egg expulsion year around. Although we did not evaluate the potential negative effects of parasites on larval fish development, the positive correlations between the PLDs of parasitized fish larvae and parasite length suggest that parasites and fish grow together and that larval parasitic copepods do not produce detrimental effects on the survival and/or development of the parasitized fish larvae. Interestingly, none of these five fish species is parasitized by pennellid copepods in their adult stages^49^ Therefore, these larval fish species, mainly *H. cunninghami* and *G. marmoratus*, could play important roles as intermediate hosts for parasitic copepods in the coastal ecosystem of the HCS.

## Supplementary Information

Supplementary Information.
