# Supplementary material for: Modelling seasonal patterns of larval fish parasitism in two northern nearshore areas in the Humboldt Current System
Source: Sci Rep. 2021 Jan 12;11:579. doi: 10.1038/s41598-020-79847-1 (PMC7804093; doi:10.1038/s41598-020-79847-1)
Supplement: Supplementary file 1 — Supplementary Information. [file 41598_2020_79847_MOESM1_ESM.docx]

**Modelling seasonal patterns of larval fish parasitism in two northern nearshore areas in the Humboldt Current System.**

Lissette D. Paredes^1,2^; Mauricio F. Landaeta^3^; Carlos Molinet^4^; M. Teresa González^2*^

^1^ Programa de Magíster en Ecología de Sistema Acuáticos, Facultad de Ciencias del Mar y Recursos Biológicos, Universidad de Antofagasta, Avenida Universidad de Antofagasta 02800, Antofagasta, Chile

^2^ Instituto de Ciencias Naturales AvH, Facultad de Ciencias del Mar y Recursos Biológicos, Universidad de Antofagasta, Avenida Universidad de Antofagasta 02800, Antofagasta, Chile

^3^ Facultad de Ciencias del Mar y de Recursos Naturales, Universidad de Valparaíso, Avenida Borgoño 16344, Reñaca, Viña del Mar, Chile

^4^ Instituto de Acuicultura. Universidad Austral de Chile, Pelluco s/n, Puerto Montt. Chile

* Corresponding author: email address: [teresa.gonzalez@uantof.cl](mailto:teresa.gonzalez@uantof.cl); ORCID:0000-0001-5787-4364

**Table S1**: Abundances of larval fish species per area (Isla Santa María and Punta Coloso) and season (Spring-Summer (SS) and Autumn-Winter (AW)). N= Number of larval fish; %= relative abundance.

|  | |  |  | |  |  | | | | |  | |  | | | | |  | | |  | | | | | |  |
| --- | --- | --- | --- | --- | --- | --- | --- | --- | --- | --- | --- | --- | --- | --- | --- | --- | --- | --- | --- | --- | --- | --- | --- | --- | --- | --- | --- |
| Family | | Species | | | Total  Abundance | | |  | | Isla Santa María | |  | | |  | |  | | | Punta Coloso | | | |  | |  |  |
|  |  |  |  |  |  |  |  | SS | | | AW | | | | |  | | SS | | | | | AW | | |  |  |
|  |  |  |  |  |  |  |  | N | % | | N | | % | | |  | | N | | | % | | N | | % | |  |
| Engraulidae | | *Engraulis ringens* | | | 21,146 | | | 113 | 0,54 | | 32 | | 0,2 | | |  | | 1635 | | | 7,7 | | 19366 | | 91,6 | |  |
| Clupeidae | | *Strangomera bentincki* | | | 20 | | | - | - | | - | | - | | |  | | - | | | - | | 20 | | 100 | |  |
| Myctophidae | | *Diogenichthys laternatus* | | | 2 | | | - | - | | - | | - | | |  | | 2 | | | 100 | | - | | - | |  |
|  | | *Hygophum bruuni* | | | 47 | | | - | - | | 1 | | 2,1 | | |  | | - | | | - | | 46 | | 97,9 | |  |
|  | | *Lampanyctus iselinoides* | | | 3 | | | - | - | | - | | - | | |  | | - | | | - | | 3 | | 100 | |  |
|  | | *Lampanyctus parvicauda* | | | 4 | | | - | - | | - | | - | | |  | | - | | | - | | 4 | | 100 | |  |
|  | | *Triphoturus oculeus* | | | 14 | | | - | - | | - | | - | | |  | | - | | | - | | 14 | | 100 | |  |
|  | | Myctophidae | | | 2 | | | 1 | 50 | | - | | - | | |  | | - | | | - | | 1 | | 50 | |  |
| Merlucciidae | | *Merluccius gayi* | | | 38 | | | 36 | 94,7 | | - | | - | | |  | | - | | | - | | 2 | | 5,3 | |  |
| Ophidiidae | | *Genypterus* sp. | | | 22 | | | 1 | 4,5 | | - | | - | | |  | | - | | | - | | 21 | | 95,5 | |  |
| Gobiesocidae | | *Gobiesox marmoratus* | | | 796 | | | 41 | 5,2 | | 305 | | 38,3 | | |  | | 102 | | | 12,8 | | 348 | | 43,7 | |  |
|  | | *Sicyases sanguineus* | | | 79 | | | - | - | | 9 | | 11,4 | | |  | | 2 | | | 2,4 | | 68 | | 86,1 | |  |
| Atherinopsidae | | *Odontesthes regia* | | | 39 | | | 1 | 2,6 | | 38 | | 97,44 | | |  | | - | | | - | | - | | - | |  |
| Sebastidae | | *Sebastes oculatus* | | | 202 | | | 21 | 10,39 | | 117 | | 57,9 | | |  | | 2 | | | 0,9 | | 62 | | 30,7 | |  |
| Paralichthyidae | | *Hippoglossina macrops* | | | 50 | | | 1 | 2 | | 6 | | 12 | | |  | | 1 | | | 2 | | 42 | | 84 | |  |
|  | | *Paralichthys adspersus* | | | 76 | | | - | - | | - | | - | | |  | | 5 | | | 6,6 | | 71 | | 93,4 | |  |
|  | | *Paralichthys microps* | | | 11 | | | - | - | | - | | - | | |  | | - | | | - | | 11 | | 100 | |  |
| Syngnathidae | | *Leptonotus blainvilleanus* | | | 4 | | | 1 | 25 | | 1 | | 25 | | |  | | 2 | | | 50 | | - | | - | |  |
| Kyphosidae | | *Girella laevifrons* | | | 209 | | | 92 | 44,02 | | 94 | | 44,9 | | |  | | 23 | | | 11,0 | | - | | - | |  |
|  | | *Graus nigra* | | | 17 | | | - | - | | - | | - | | |  | | - | | | - | | 17 | | 100 | |  |
| Pomacentridae | | *Chromis crusma* | | | 52 | | | 4 | 7,7 | | - | | - | | |  | | 28 | | | 53,9 | | 20 | | 38,5 | |  |
| Nomeidae | | *Cubiceps sp.* | | | 2 | | | - | - | | - | | - | | |  | | 2 | | | 100 | | - | | - | |  |
| Stromateidae | | *Stromateus stellatus* | | | 1 | | | - | - | | - | | - | | |  | | - | | | - | | 1 | | 100 | |  |
| Pinguipedidae | | *Pinguipes chilensis* | | | 48 | | | - | - | | - | | - | | |  | | - | | | - | | 48 | | 100 | |  |
|  | | *Prolatilus jugularis* | | | 56 | | | - | - | | - | | - | | |  | | - | | | - | | 56 | | 100 | |  |
| Clinidae | | *Myxodes spp* | | | 262 | | | 7 | 2,7 | | 255 | | 97,3 | | |  | | - | | | - | | - | | - | |  |
| Labrisomidae | | *Auchenionchus crinitus* | | | 8,231 | | | 113 | 1,4 | | 7704 | | 93,6 | | |  | | 14 | | | 0,2 | | 400 | | 4,9 | |  |
|  | | *Auchenionchus microcirrhis* | | | 2,373 | | | 1 | 0,04 | | 478 | | 20,1 | | |  | | - | | | - | | 1894 | | 79,8 | |  |
|  | | *Calliclinus genigutattus* | | | 756 | | | 17 | 2,3 | | 724 | | 95,8 | | |  | | 1 | | | 0,1 | | 14 | | 1,9 | |  |
|  | | *Labrisomidae* | | | 34 | | | - | - | | 6 | | 17,6 | | |  | | 10 | | | 29,4 | | 18 | | 52,9 | |  |
| Gobiidae | | *Ophiogobius jenynsi* | | | 411 | | | - | - | | 342 | | 83,2 | | |  | | - | | | - | | 69 | | 16,8 | |  |
| Tripterygiidae | | *Helcogrammoides cunninghami* | | | 9,833 | | | 106 | 1,8 | | 9669 | | 98,3 | | |  | | 3 | | | 0,03 | | 55 | | 0,6 | |  |
| Dactyloscopidae | | *Sindoscopus australis* | | | 94 | | | 3 | 3,2 | | 50 | | 53,2 | | |  | | - | | | - | | 41 | | 43,6 | |  |
| Blenniidae | | *Hypsoblennius sordidus* | | | 376 | | | 12 | 3,2 | | 202 | | 53,7 | | |  | | 64 | | | 17,0 | | 98 | | 26,1 | |  |
|  | | *Scartichthys viridis* | | | 2,154 | | | 842 | 39,09 | | 856 | | 39,7 | | |  | | 342 | | | 15,9 | | 114 | | 5,3 | |  |
| Normanichthyidae | | *Normanichthys crockeri* | | | 132 | | | 3 | 2,3 | | 2 | | 1,5 | | |  | | 3 | | | 2,3 | | 124 | | 93,9 | |  |
| Agonidae | | *Agonopsis chiloensis* | | | 12 | | | 11 | 91,66 | | 1 | | 8,3 | | |  | | - | | | - | | - | | - | |  |
| Sciaenidae | | Sciaenidae | | | 20 | | | 4 | 20 | | - | | - | | |  | | 6 | | | 30 | | 10 | | 50 | |  |
| Total abundace | |  | | | 47,628 | | | 1,431 | | | 20,892 | | | | |  | | 2,247 | | | | | 23,058 | | | |  |
| Total taxa | |  | | | 38 | | | 22 | | | 21 | | | | |  | | 19 | | | | | 31 | | | |  |
|  | |  | | |  | | |  |  | |  | |  | | |  | |  | | |  | |  | |  | |  |
|  | |  | | |  | | |  |  | |  | |  | | |  | |  | | |  | |  | |  | |  |
|  | |  | | |  | | |  |  | |  | |  | | |  | |  | | |  | |  | |  | |  |
|  | |  | | |  | | |  |  | |  | |  | | |  | |  | | |  | |  | |  | |  |
|  | |  | | |  | | |  |  | |  | |  | | |  | |  | | |  | |  | |  | |  |
|  | |  | | |  | | |  |  | |  | |  | | |  | |  | | |  | |  | |  | |  |
|  | |  | | |  | | |  |  | |  | |  | | |  | |  | | |  | |  | |  | |  |
|  | |  | | |  | | |  |  | |  | |  | | |  | |  | | |  | |  | |  | |  |
